# Stimulation of EphB2 attenuates tau phosphorylation through PI3K/Akt-mediated inactivation of glycogen synthase kinase-3β

**DOI:** 10.1038/srep11765

**Published:** 2015-06-29

**Authors:** Jun Jiang, Zhi-Hao Wang, Min Qu, Di Gao, Xiu-Ping Liu, Ling-Qiang Zhu, Jian-Zhi Wang

**Affiliations:** 1 Department of Pathophysiology, School of Basic Medicine and the Collaborative Innovation Center for Brain Science, Key Laboratory of Neurological Diseases of Education Ministry of China, Tongji Medical College, Huazhong University of Science and Technology, Wuhan, P.R. China; 2 Hubei Provincial Center for Disease Control and Prevention, Wuhan, P. R. China; 3 Department of Oncology, The Central Hospital of Wuhan, 430014, Wuhan, China; 4 Clinical Laboratory of Hangzhou Traditional Chinese Medical Hospital, Hangzhou, P. R. China; 5 Co-innovation Center of Neuroregeneration, Nantong University, Nantong, JS 226001, China

## Abstract

Abnormal tau hyperphosphorylation is an early pathological marker of Alzheimer’s disease (AD), however, the upstream factors that regulate tau phosphorylation are not illustrated and there is no efficient strategy to arrest tau hyperphosphorylation. Here, we find that activation of endogenous EphB2 receptor by ligand stimulation (ephrinB1/Fc) or by ectopic expression of EphB2 plus the ligand stimulation induces a remarkable tau dephosphorylation at multiple AD-associated sites in SK-N-SH cells and human embryonic kidney cells that stably express human tau (HEK293-tau). In cultured hippocampal neurons and the hippocampus of human tau transgenic mice, dephosphorylation of tau proteins was also detected by stimulation of EphB2 receptor. EphB2 activation inhibits glycogen synthase kinase-3β (GSK-3β), a crucial tau kinase, and activates phosphatidylinositol-3-kinase (PI3K)/Akt both *in vitro* and *in vivo*, whereas simultaneous inhibition of PI3K or upregulation of GSK-3β abolishes the EphB2 stimulation-induced tau dephosphorylation. Finally, we confirm that ephrinB1/Fc treatment induces tyrosine phosphorylation (activation) of EphB2, while deletion of the tyrosine kinase domain (VM) of EphB2 eliminates the receptor stimulation-induced GSK-3β inhibition and tau dephosphorylation. We conclude that activation of EphB2 receptor kinase arrests tau hyperphosphorylation through PI3K-/Akt-mediated GSK-3β inhibition. Our data provide a novel membranous target to antagonize AD-like tau pathology.

Hyperphosphorylation of microtubule associated protein tau is an early pathological marker of Alzheimer’s disease (AD)^1^. Upon hyperphosphorylation, tau proteins are polymerized into paired helical filaments (PHF) and neurofibrillary tangles^2^,^3^. The hyperphosphorylated tau is incompetent in promoting microtubule assembly and maintaining the stability of microtubules that consequently lead to destruction of the neuronal cytoskeleton and axonal transport^4^,^5^,^6^. It has been demonstrated that the abnormal tau-related neurodegeneration is positively correlated with the degree of dementia^7^,^8^, while there is no efficient approach to arrest tau pathologies, to date.

Several protein kinases have been reported to play a role in AD-like tau hyperphosphorylation, among them, glycogen synthase kinase-3β (GSK-3β) is the most implicated^9^. GSK-3β is the first identified tau kinase^10^, which can phosphorylate tau at multiple AD-associated sites and thus inhibits the biological activity of tau in promoting microtubule assembly and stabilizing microtubules^11^. The active form of GSK-3β is co-localized with the neurofibrillary tangles in the AD brains^12^. Stimulation of GSK-3β both *in vitro* and *in vivo* induces tau hyperphosphorylation with impairments of the cognitive functions, whereas inhibition of GSK-3β improves tau pathologies and memory deficits^13^,^14^,^15^,^16^. Activation of GSK-3β inhibits long-term potentiation (LTP) and impairs synaptic function^17^,^18^,^19^, which may underlie the GSK-3β-induced memory deficits. GSK-3β also plays a crucial role in tau exon 10 splicing^20^. Several intracellular pathways have been identified to regulate GSK-3β activity, such as PI3K/Akt and protein kinase C (PKC) pathways^13^,^21^. However, it is not fully illustrated whether and how the plasma membraneous receptors, the best accessible drug targets, may regulate tau phosphorylation through GSK-3β.

Eph receptor is a member of receptor tyrosine kinases (RTKs) that play a critical role in the development of the central nervous system^22^,^23^,^24^. The Eph receptors and their ephrin ligands are divided into two subsets, i.e., ephrinA and ephrinB. In general, the EphA receptors bind promiscuously to glycosyl-phosphatidyl-inisotol (GPI)-anchored ephrinA ligands, while the EphB receptors interact with transmembrane ephrinB ligands. As a member of EphB family, EphB2 and the ligand ephrinB1 are highly expressed in the adult nervous system^25^,^26^, where the receptor plays a crucial role in synaptic functions and synaptopathies, such as regulating synaptic plasticity, enhancing dendritic filopodia motility and promoting axon growth and regeneration^22^,^23^,^24^,^27^. EphB2 shows an age- and brain region-dependent reduction, and it is translocated into the intracellular compartment when exposed to Aβ^28^. A reduction of EphB2 receptor was observed in the hippocampus of AD patients at an incipient stage and in AD transgenic mice^29^. A recent study also demonstrated that knockdown of EphB2 in mice by shRNA reduced N-methyl-D-aspartate receptor (NMDAR) currents and impaired long-term potentiation in the dentate gyrus, while increasing EphB2 expression in the dentate gyrus of human amyloid precursor protein transgenic mice reversed memory deficits^30^. Interaction of Eph-ephrin activates the receptor and triggers cytoskeleton remodeling^31^. Tau is a major cytoskeleton protein that is hyperphosphorylated in the AD brains, but it is currently not known whether EphB2/ephrinB1 regulates phosphorylation of tau proteins.

In the present study, we activate the endogenous EphB2 receptor in SK-N-SH cells, mouse hippocampal neuron culture and human tau transgenic mice by using ephrinB1/Fc (the chimeric agonist of EphB2), or by ectopically expressing EphB2 with application of ephrinB1/Fc in HEK293-tau cells that do not express endogenous EphB2. Then we measured the phosphorylation level of tau and the GSK-3β-related signaling pathway. We demonstrate that activation of EphB2 induces tau dephosphorylation at multiple AD-related sites with mechanisms involving the EphB2 kinase-coupled PI3K/Akt activation and GSK-3β inhibition.

## Results

### Stimulation of EphB2 attenuates tau phosphorylation both *in vitro* and in hippocampus of human tau transgenic mice

**Figure 1:**
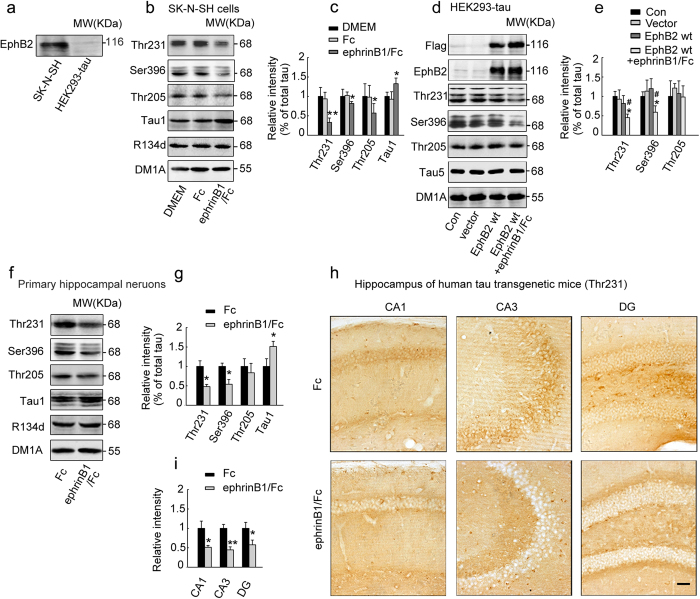
Stimulation of EphB2 induces tau dephosphorylation in cells and hippocampus of human tau transgenic mice. (**a**) SK-N-SH but not HEK293-tau cells express endogenous EphB2 receptor measured by Western blotting. (**b, c**) EphB2 was stimulated by treatment of the SK-N-SH cells with ephrinB1/Fc dissolved in DMEM for 45 min, and then tau phosphorylation at different sites as labeled was measured by Western blotting. (**d, e**) The flag-labeled wild type EphB2 (EphB2wt) plasmid was transfected into HEK293-tau cells for 24 h and then the cells were treated with ephrinB1/Fc for 45 min before detection of tau phosphorylation. (**f, g**) The primary hippocampal neurons (cultured for 7 days *in vitro*) were treated with ephrinB1/Fc for 45 min, and then tau phosphorylation was measured by Western blotting. (**h, i**) The ephrinB1/Fc was infused into hippocampal CA3 region of the human tau transgenic mice (10 m old) for 45 min, and then tau phosphorylation in hippocampus was measured by immunohistochemistry staining (**h**) and quantitative analysis (**i**). Scale bar = 20 μm. Dephosphorylation of tau was detected by stimulation of EphB2 receptor. The total tau was probed by R134d or Tau5 that was normalized against tubulin (DM1A), and the phosphorylation level of tau at Thr205, Thr231, Ser396 and tau-1 sites was normalized against total tau. [Note that tau-1 reacts with the unphosphorylated tau at Ser198/199/202, therefore an increased immunoreaction to tau-1 suggests an increased tau dephosphorylation]. The blots were representative of at least three independent experiments with cells from 9 ~ 12 culture wells for each group. The images were representative of at least 3 mice. *P < 0.05, **P < 0.01 *versus* Fc or DMEM or vector; #*P* < 0.05 *versus* EphB2 wt alone (mean±SD).

By Western blotting, we show that SK-N-SH cells express endogenous EphB2 while HEK293 cells with stable express of exogenous human full length tau (HEK293-tau) do not express EphB2 (Fig. 1a). By treated SK-N-SH cells with ephrinB1/Fc, a chimeric activator of EphB2, we find that activation of EphB2 attenuates tau phosphorylation at Thr205, Thr231, Ser396, and tau-1 epitope with a time dependent manner, and the dephosphorylation was most significant at 30 min and 45 min (Fig. 1b,c; and Supplementary Fig. 1). Therefore we chose 30 min or 45 min for ephrinB1/Fc treatment in the remaining studies. Tau dephosphorylation at Thr231 and Ser396 was also detected in SK-N-SH cells by immunofluorescence staining after ephrinB1/Fc compared with Fc alone (Supplementary Fig. 2). Further studies show that exogenous expression of EphB2 plus ephrinB1/Fc stimulation but not EphB2 alone can also attenuate tau phosphorylation in HEK293-tau cells that do not have endogenous EphB2 system (Fig. 1d,e). In primary hippocampal neurons, stimulating EphB2 also induces tau dephosphorylation (Fig. 1f,g). To explore the *in vivo* effects of EphB2 on tau phosphorylation, we injected ephrinB1/Fc into the hippocampal CA3 region of 10 m-old human tau transgenic mice for 45 min and then measured tau phosphorylation level. The results show that *in vivo* stimulation of EphB2 also reduces tau phosphorylation in hippocampal CA1, CA3 and dentate gyrus (DG) shown by immunohistochemistry (Fig. 1h,i) and immunofluorescence staining (Supplementary Fig. 3). These data together suggest that stimulation of EphB2 attenuates tau phosphorylation at multiple AD-associated sites both *in vitro* and *in vivo*.

### Stimulation of EphB2 inhibits GSK-3β activity

**Figure 2:**
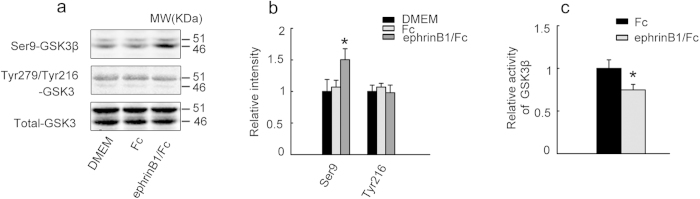
Stimulation of EphB2 inhibits GSK-3β. (**a, b**) SK-N-SH cells were treated with ephrinB1/Fc or Fc or DMEM (vehicle control) for 45 min to activate EphB2 and then the activity-dependent phosphorylation of GSK-3β at Ser9 (inactivation) and Tyr216 (activation) was measured by Western blotting (**a**) and quantitative analysis (**b**). (**c**) The inactivation of GSK-3β was also observed by biochemical assay of the kinase activity. Data were representative of at least three independent experiments with cells from 9~12 culture wells for each group and presented as the mean±SD. *P < 0.05 *versus* Fc or DMEM.

To explore the mechanisms underlying the EphB2-induced tau dephosphorylation, we measured the alterations of GSK-3β, the most implicated kinase in regulating tau phosphorylation^9^. We found that stimulation of EphB2 by treatment of SK-N-SH cells with ephrinB1/Fc for 30 min significantly increased the level of Ser9-GSK-3β (the inactivated form) with no effect on Tyr216-GSK-3 (the active form) and total-GSK-3 (Fig. 2a,b). To confirm the effect of EphB2 stimulation on GSK-3β, we measured the activity of GSK-3β by chemical assays. The data show that stimulation of EphB2 by ephrinB1/Fc inhibits GSK-3β activity (Fig. 2c). These data suggest that stimulation of EphB2 inhibits GSK-3β.

### Stimulation of EphB2 upregulates PI3K and Akt with inhibition of GSK-3β

**Figure 3:**
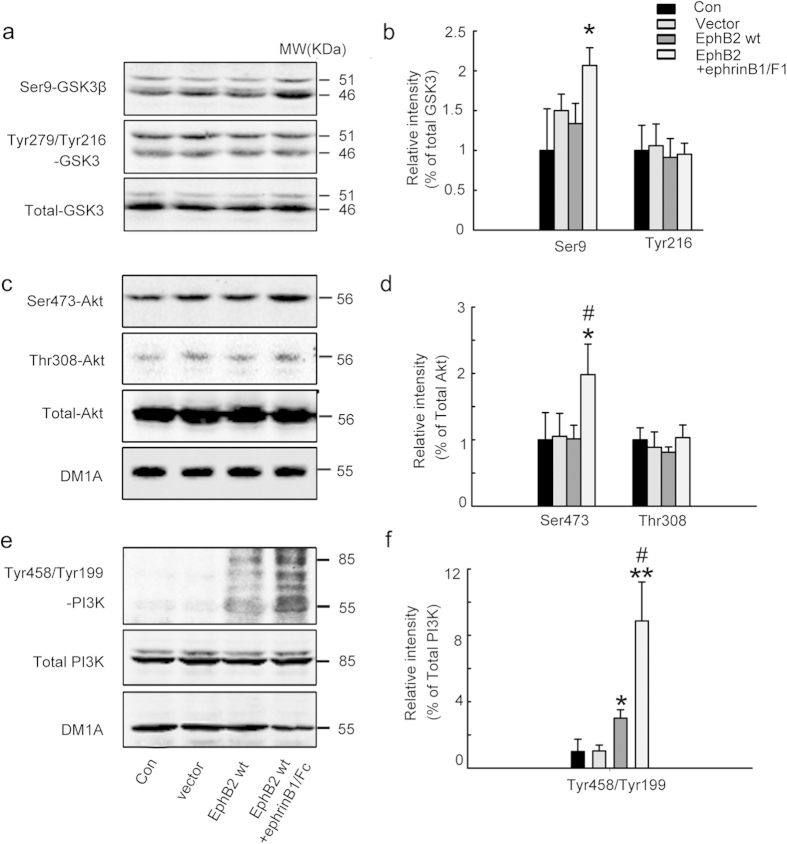
Stimulation of EphB2 up-regulates PI3K and Akt with GSK-3β inhibition. HEK293-tau cells were untreated (Con) or transfected with pcDNA3.0 (Vector) or transfected with wild type EphB2 alone (EphB2wt, inactive) for 24 h, or transfected with EphB2wt for 24 h and then stimulated with ephrinB1/Fc for 45 min. The activity-dependent phosphorylation of GSK-3β at Ser9 (inactive form) and Tyr216 (active form) (**a, b**), Akt at Ser473 and Thr308 (active form) (**c, d**), and PI3K at Tyr458/199 (active form) (e, f) was measured respectively by Western blotting and quantitative analysis. The phosphorylation level of the kinases was normalized against the total level. Data were representative of at least three independent experiments with cells from 9~12 culture wells for each group and presented as the mean ± SD. *P < 0.05, **P < 0.01 *versus* Con and Vector; #*P* <0.05 *versus* EphB2 wt.

The activity of GSK-3β is regulated by PI3K/Akt^21^, therefore, we measured the activity-dependent phosphorylation of PI3K and Akt after transfection of EphB2 alone (inactive EphB2) or EphB2 plus treatment of ephrinB1/Fc (activated EphB2) in HEK293-tau cells. Accompany by an increased phosphorylation of GSK-3β at Ser9 (inactivation) in ephrinB1/Fc group (Fig. 3a,b), the phosphorylation levels of PI3K at Tyr458/199 (active form) and of Akt at Ser473 (active form) were significantly increased (Fig. 3c–f). These data suggest that PI3K/Akt activation mediates the EphB2-induced GSK-3β inhibition.

### PI3K/Akt-mediated GSK-3β inhibition is required for the EphB2 stimulation-induced tau dephosphorylation

**Figure 4:**
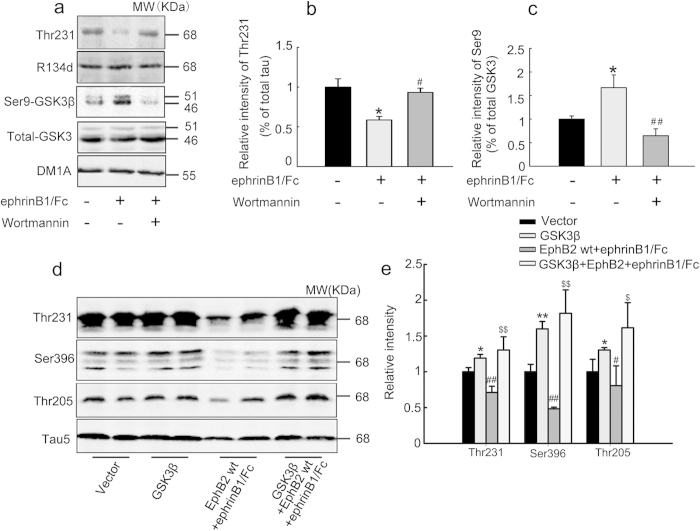
Simultaneous inhibition of PI3K or overexpression of GSK-3β abolishes the EphB2 activation-induced tau dephosphorylation. (**a-c**) SK-N-SH cells were treated with ephrinB1/Fc alone or ephrinB1/Fc plus wortmannin (an inhibitor of PI3K) for 2 h, and then phosphorylation level of GSK-3β at Ser9 and tau at Thr231 was measured by Western blotting. *P < 0.05 *versus* untreated control; #*P* < 0.05, ##*P* < 0.01 *versus* ephrinB1/Fc. (**d, e**) HEK293 cells were transfected with pcDNA3.0 (Vector), or with GSK-3β, or with EphB2wt for 24 h and ephrinB1/Fc stimulation for 45 min, or with co-transfection of EphB2wt and GSK-3β□plus ephrinB1/Fc stimulation. The phosphorylation levels of GSK-3β at Ser9 and tau at Thr205, Thr231 and Ser396 were measured by Western blotting. The phosphorylation level of GSK-3β at Ser9 was normalized against total GSK-3β, while the phosphorylation of tau was normalized against total tau probed by R134d or Tau5. Data were representative of at least three independent experiments with cells from 9 ~ 12 culture wells for each group and presented as the mean ± SD. *P < 0.05, **P < 0.01 GSK-3β *versus* Vector; #*P* < 0.05, ##*P* < 0.01 EphB2/ephrinB1/Fc *versus* GSK-3β; $ <0.05, $$ *P* < 0.01 GSK-3β/EphB2/ephrinB1/Fc *versus* EphB2/ephrinB1/Fc.

To further verify the role of PI3K/Akt-mediated GSK-3β inhibition in EphB2 stimulation-induced tau dephosphorylation, we first used wortmannin, a PI3K inhibitor, in SK-N-SH cells. We found that simultaneous inhibition of PI3K almost abolished the ephrinB1/Fc-induced GSK-3β phosphorylation at Ser9 and tau dephosphorylation (Fig. 4a–c), suggesting that PI3K/Akt plays a crucial role in ephrinB1/Fc-induced GSK-3β inhibition and tau dephosphorylation. To further confirm the role of GSK-3β inhibition in EphB2-induced tau dephosphorylation, we simultaneously transfected GSK-3β with EphB2 plasmids into HEK293-tau cells before ephrinB1/Fc treatment. We observed that simultaneous upregulation of GSK-3β abolished EphB2 stimulation-induced tau dephosphorylation at Ser396, Thr231 and Thr205 sites (Fig. 4d,e), which confirm the role of GSK-3β inhibition in EphB-induced tau dephosphorylation.

**Figure 5:**
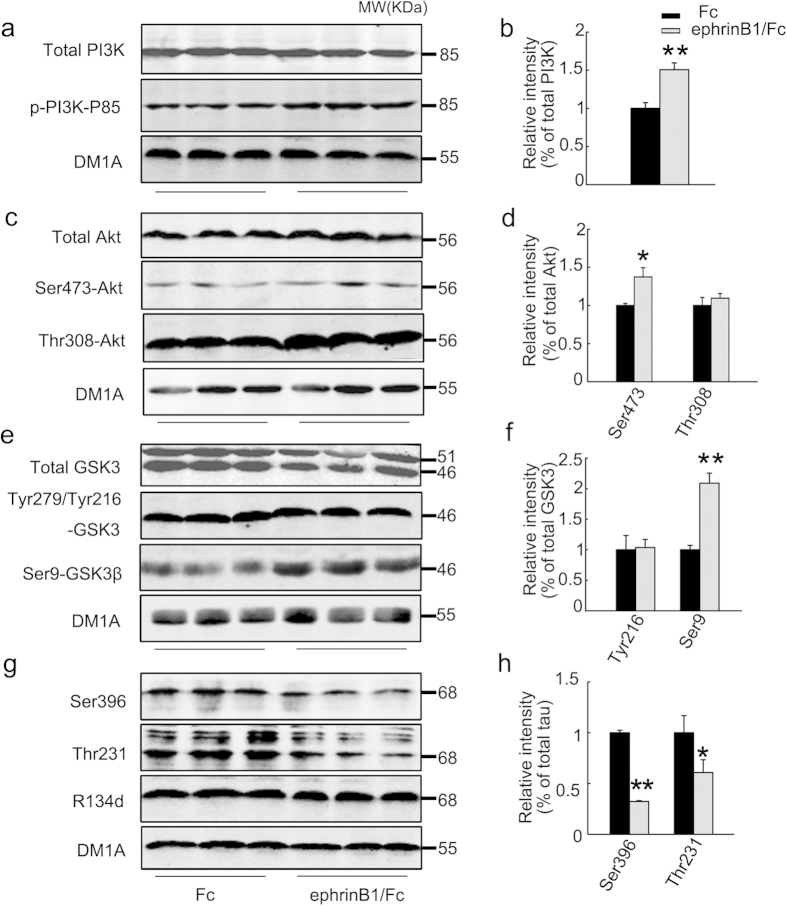
Stimulation of EphB2 induces tau dephosphorylation with activation of PI3K/Akt and inhibition of GSK-3 in hippocampus of human tau transgenic mice. EphrinB1/Fc or Fc was infused stereotactically into the hippocampal CA3 region of the human tau transgenic mice for 45 min and then the total and the activity-dependent phosphorylation levels of PI3K (**a, b**), Akt (**c, d**), GSK-3 (**e, f**), and the phosphorylation level of tau (**g, h**) were measured by Western blotting. The phosphorylation level of the kinases was normalized against total kinase level, while the phosphorylation level of tau was normalized against total tau probed by R134d. The total levels of the kinases and tau were normalized against tubulin probed by DM1A. The blots were representative of at least three independent experiments from at least 6 mice for each group and presented as the mean ± SD. *P < 0.05, **P < 0.01 *versus* Fc.

To explore the *in vivo* effects of EphB2 stimulation in tau dephosphorylation and the involvement of PI3K/Akt/GSK-3β pathway, we infused ephrinB1/Fc or Fc as control into the hippocampal CA3 region of the human tau transgenic mice and then measured the alterations of tau phosphorylation and PI3K/Akt/GSK-3β signaling pathway. We observed that stimulation of EphB2 by ephrinB1/Fc increased phosphorylation (activation) of PI3K at P85 and Akt at Ser473 with a simultaneous inactivation of GSK-3β and dephosphorylation of tau at Ser396 and Thr231 (Fig. 5).

These *in vitro* and *in vivo* data together confirm the role of PI3K/Akt-mediated GSK-3β inactivation in EphB2-induced tau dephosphorylation.

### EphrinB1/Fc stimulates EphB2 tyrosine phosphorylation and deletion of the kinase domain eliminates EphB2-induced tau dephosphorylation

**Figure 6:**
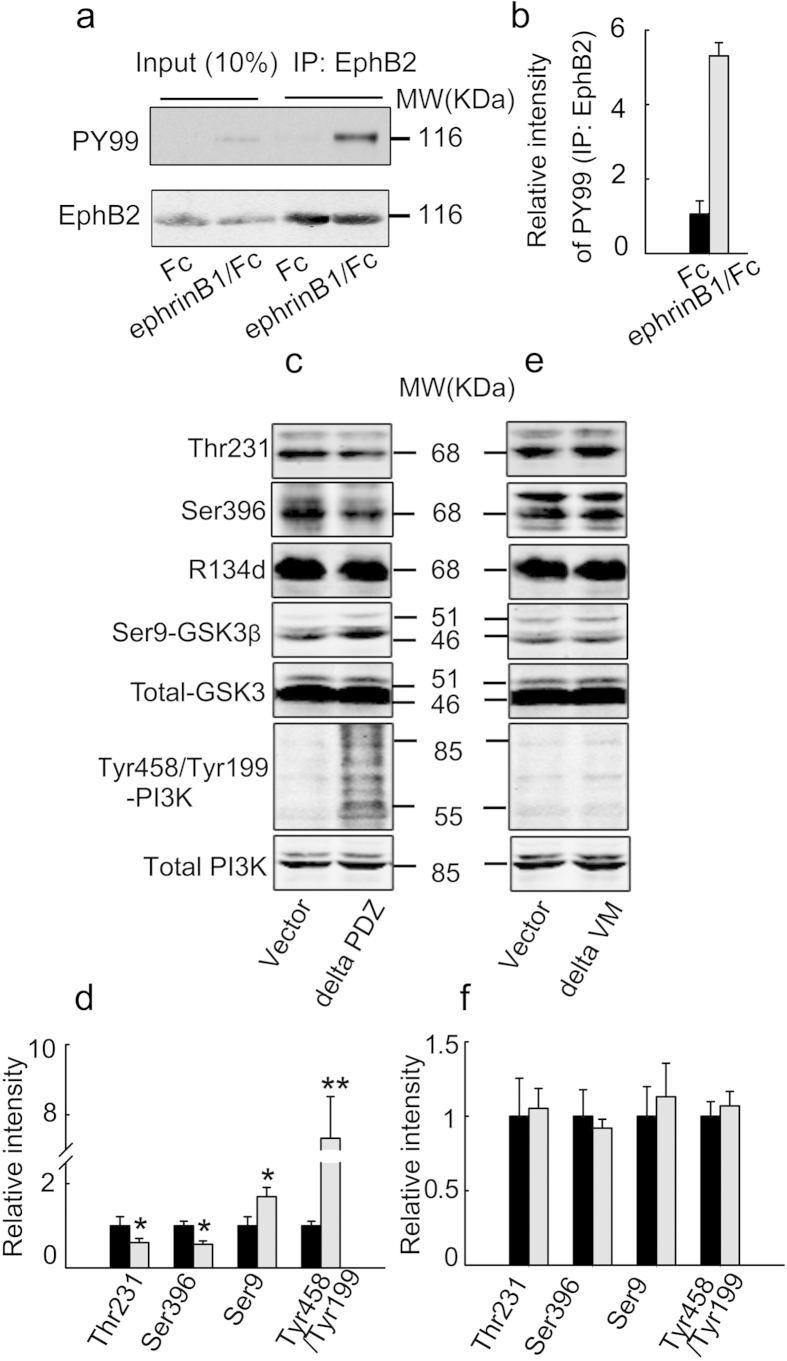
EphrinB1/Fc stimulates tyrosine phosphorylation of EphB2 and deletion of the kinase domain (VM) but not the PDZ binding domain eliminates EphB2-induced tau dephosphorylation. HEK293-tau cells were treated with ephrinB1/Fc for 45 min, and then the phosphorylation level of EphB2 was measured by immunoprecipitation with anti-EphB2 antibody and Western blotting probed by PY99 and anti-EphB2 antibody (**a, b**). HEK293-tau cells were transfected with deletion of PDZ (**c, d**) or VM (**e, f**) domain of EphB2 or the control Vector for 24 h and then treated with ephrinB1/Fc for 45 min. The phosphorylation levels of tau at Thr231 and Ser396, or GSK-3β at Ser9, or PI3K at Tyr458/Tyr199 were measured by Western blotting. The phosphorylation levels of tau and the kinases were normalized against the total levels. Data were representative of at least three independent experiments from 9 ~ 12 culture wells for each group and presented as the mean ± SD. *P < 0.05, **P <0.01 *versus* Vector.

The activity of tyrosine kinases is stimulated by autophosphorylation within the kinase domain^32^,^33^. To confirm the receptor activation, we measured the tyrosine phosphorylation by EphB2 immunoprecipitation and P-Tyr-99 blotting in SK-N-SH cells after treatment of ephrinB1/Fc for 45 min. The results showed that ephrinB1/Fc treatment increased remarkably the phosphorylation level of EphB2 (Fig. 6a,b), indicating the kinase activation. Both the kinase domain (VM) and PDZ binding domain are important functional regions of EphB2 receptor. To further confirm the role of EphB2 kinase domain and possible involvement of PDZ in regulating PI3K/ GSK-3β pathway and tau dephosphorylation, we transfected EphB2 VM or PDZ deletion mutant plasmids into the HEK293-tau cells and treated the cells with ephrinB1/Fc for 45 min. We found that deletion of PDZ domain did not affect the EphB2 stimulation-induced phosphorylation levels of PI3K/GSK-3β and tau proteins (Fig. 6c,d), whereas deletion of VM kinase domain almost abolished the EphB2 activation-induced alterations of PI3K/GSK-3β and tau phosphorylation (Fig. 6e,f). These data suggest that the tyrosine phosphorylation and the kinase domain (VM) of EphB2 play a key role in coupling PI3K/GSK-3β pathway with the reduced tau phosphorylation.

## Discussion

AD is the most common cause of senile dementia affecting an increased number of the elder populations. With a worldwide population aging, the incidence of AD is increasing and there is an urgent need of effective cure for this devastating disorder. Intracellular accumulation of the hyperphosphorylated tau forming neurofibrillary tangles is one of the two pathological hallmarks in the AD brains, and the amount of tangles is positively correlated with the degree of dementia^7^,^8^. Therefore, searching for upstream factors that can attenuate the AD-like tau hyperphosphorylation is of great importance in designing efficient therapies for AD. In the present study, we investigated whether EphB2, a receptor tyrosine kinase, is involved in tau phosphorylation and the underlying signaling pathway.

We first investigated whether neuroblastoma SK-N-SH cells and HEK293-tau express endogenous EphB2. The human embryonic kidney cells do not express endogenous tau but with stable expression of human tau441 established in our laboratory^34^,^35^. EphB2 was only detected in SK-N-SH cells but not in HEK293-tau cells. Recent studies demonstrate that EphB2 receptors are expressed in many cell types, including neurons in the brains of rat^36^ and mouse^37^, and in abdominal aortic aneurysm of human^38^. Our current study demonstrates that SK-N-SH cells also express EphB2. To activate the EphB2 in SK-N-SH cells, the cultured hippocampal neurons or in the brain of the human tau transgenic mice, we employed ephrinB1/Fc, the chimeric ligand stimulator of EphB2. We found that stimulation of endogenous EphB2 by ephrinB1/Fc could induce tau dephosphorylation at multiple AD-associated phosphorylation sites. Since HEK293-tau cells do not express endogenous EphB2, we treated this cell line by transfected the exogenous EphB2 with stimulation of ephrinB1/Fc, and then measured tau phosphorylation level. A remarkable tau dephosphorylation was also detected. These *in vitro* and *in vivo* data demonstrate that stimulation of EphB2 can attenuate tau phosphorylation.

Tau phosphorylation is regulated by protein kinases and protein phosphatases, among various kinases, GSK-3β activation is one of the most implicated in the AD-like tau hyperphosphorylation^9^. Therefore, we measured whether stimulation of EphB2/ephrinB1 signaling affects GSK-3β. By measuring directly GSK-3β activity or the activity-dependent phosphorylation of GSK-3β at Ser9 (inhibitory) and Tyr216 (activation)^39^, we found that stimulation of EphB2 inhibits GSK-3β. To confirm the role of GSK-3β inhibition in EphB2/ephrinB1-induced tau dephosphorylation, we used wortmannin (a PI3K inhibitor that can indirectly activate GSK-3β)^40^ or overexpression of wild type GSK-3β. The results show that simultaneous activation of GSK-3β abolishes the EphB2 stimulation-induced tau dephosphorylation. These data together strongly support that suppression of GSK-3β is responsible for the EphB2-induced tau dephosphorylation. A previous study demonstrates that activation of GSK-3β can down-regulate EphB2/ephrinB1 in intestinal epithelial cells^41^, while our current paper is the first to show that stimulation of EphB2/ephrinB1 inactivates GSK-3β. These results suggest a feedback regulation between GSK-3β and EphB2/ephrinB1.

Interaction of Ephs and the ligand ephrins initiates distinct, bidirectional signaling pathways known as forward (Eph) and reverse (ephrin) signaling. Like other Eph receptors, the monomer form of EphB2 receptor has no activity, and association of EphB2 with its ephrin ligands activates the receptor. The ephrinB ligands can be divided into three subgroups, namely B1, B2 and B3. We choose to use ephrinB1 because it is the most abundantly expressed form in the brain^26^. Furthermore, the soluble form ephrinB1 is inactive and it is activated by fused with human IgG to form ephrinB1/Fc clusters^42^. We also observed in the present study that no stimulating effects were detected by treatment of ephrinB1 or Fc alone. The activation of EphB2 can be evaluated by measuring the tyrosine kinase autophosphorylation^43^ or activation of the downstream signaling molecules, such as c-fos^44^. In the present study, the activation of EphB2 receptor by ephrinB1/Fc stimulation was proved by the increased tyrosine phosphorylation and activation of its downstream PI3K/Akt.

The kinase domain and the PDZ-binding domain are two major structural domains of EphB2. Upon binding, the dimerized or aggregated Eph-ephrin receptors can transduce signals via phosphorylation of the conserved tyrosine residues^23^,^45^,^46^. Regarding the role of kinase domain in mediating the EphB2 signals, conflicting results were reported, some show that the kinase domain is not required for the EphB2-mediated excitatory synaptic plasticity^26^,^47^, whereas others suggest that EphB2/CTF2, but not a kinase-deficient mutant of EphB2/CTF2, promotes the cell surface expression of N-methyl-D-aspartate receptor (NMDAR)^48^. Our results show that the kinase domain is required for the downstream signal transduction of EphB2/ephrinB1 in HEK293-tau cells. By using blue native polyacrylamide gel electrophoresis and mass spectrometry, alteration of focal adhesion kinase (FAK) and Nischarin signaling were identified in NG108 cells after stimulation of EphB2/ephrinB1^49^. We demonstrate here that stimulation of EphB2/ephrinB1 can activate PI3K/Akt signaling, which add new targets for the EphB2/ephrinB1 pathways.

Taken together, we find in the present study that stimulation of EphB2/ephrinB1 attenuates tau phosphorylation through PI3K/Akt/GSK-3β-mediated pathway. Therefore, EphB2/ephrinB1 receptor could be an upstream factor in attenuating AD-like tau pathology.

## Methods

### Chemicals and antibodies

EphB2 wt-flat, delta VM (VM domain knockout), and delta PDZ (PDZ domain knockout) plasmids were kind gifts of Prof. Matthew B. Dalva in Department of Neuroscience, University of Pennsylvania School of Medicine. The ephrinB1 and Fc were purchased from R&D Systems (Minneapolis, MN). The ephrinB1/Fc chimera or Fc control was preclustered by incubating with anti-human Fc IgG at 37 °C for 30 min following the manufacturer’s instructions, and the final concentration of ephrinB1 or Fc is 2 μg/ml. The information for antibodies employed was listed in Table 1. The wild type GSK-3β (wtGSK-3β) plasmid with HA-tag was a gift from Dr Woodgett at Toronto University, Canada.

### Animals and drug administration

Ten month-old human tau transgenic mice (Jackson lab, STOCK Mapttm1 (EGFP)Klt Tg(MAPT)8cPdav/J) were used for *in vivo* studying the effects of EphB2/ephrinB1/Fc on tau dephosphorylation. Mice were housed in the Animal Resource Center at Tongji Medical College, Huazhong University of Science and Technology. All experiments conducted on animals were performed according to protocols approved by the institutional Animal Care and Use Committee.

To study the *in vivo* effects of EphB2 stimulation on tau dephosphorylation, ten ~10 months old human tau transgenic mice, which express six isoforms of tau and show an age-dependent development tau pathology (Polydoroetal., 2009) were used. For the brain infusion, the mice were anesthetized with 6% chloral hydrate (6 ml/kg) and placed in a Jiangwan-II stereotaxic instrument (Jiangwan Medical Instrument Co. Shanghai, China). After the scalp was incised, the skull was cleaned and a hole (diameter 0.5 mm) was made for the injection. The co-ordinates of AP-2.18, L-2.6, V-2.3 (in mm from bregma) for CA3 area of hippocampus were selected according to the stereotaxic atlas of Franklin and Paxinos. A sterilized needle connected to a 5 μl syringe (Hamilton) was used to deliver ephrinB1/Fc chimera or Fc control in 1 μl to the left hippocampus of the mice, after 45 min, the mice were sacrificed for further studies.

### Cell culture and treatments

Human neuroblastoma SK-N-SH cells (Shanghai Biochemical Department, Shanghai, China) that express endogenous EphB2 receptor and tau proteins were cultured in Dulbecco’s modified eagle’s medium (DMEM) supplemented with 10% fetal bovine serum (FBS, Gibco BRL, Gaithersburg, MD) and grown at 37 °C in a humid atmosphere containing 5% carbon dioxide, and ephrinB1/Fc was incubated with the cells for 45 min to activate EphB2.

HEK293 cells that do not express endogenous tau were transfected with the longest human tau (tau441) or the pcDNA 3.0 vector, and the cell line with stable expression of tau (HEK293-tau) or the vector (HEK293/vector) were established from our group^34^. The cells were cultured in DMEM containing 10% FBS and 200 μg/ml G418. Since HEK293 cells do not express EphB2, we transfected wild type EphB2, EphB2 with delta VM or delta PDZ, or pcDNA3.1 as vector control with treatment of ephrinB1/Fc to activate EphB2. The transfection was carried out by following the manufacturer’s instructions. In brief, EphB2 plasmids or the vector (4 μg) were mixed with Lipofectamine-2000 in 250 μl OPTI-MEM followed by incubation at room temperature for 10 min. Then the Lipofectamine-DNA complex was added to HEK293-tau cells and the cells were incubated at 37 °C for further treatment. After ephrinB1/Fc treatment for 45 min, the cells were harvested for Western blotting. To inhibit PI3K, HEK293-tau cells were treated with wortmannin (1 μM, Tocris Cookson) for 1 h before administration of ephrinB1/Fc. To confirm the activation of EphB2, the phosphorylation level of EphB2 was measured by immunoprecipitation with EphB2 antibody and Western blotting using PY99 antibody (Santa Cruz)^43^.

The primary neurons from E18 rat hippocampus that express endogenous tau proteins and EphB2 receptor were seeded at 30,000–40,000 cells per well in neurobasal medium supplemented with 2% B27/0.5 mM glutamine/25 mM glutamate. Half of the culture medium was changed every 3 days with neurobasal medium. All cultures were kept in a humidified 5% CO_2_ containing atmosphere at 37 °C. After cultured for 7 days, more than 90% of the cells were neurons *in vitro*. Then the cells were harvested for Western blotting after ephrinB1/Fc treatment for 45 min.

### Immunohistochemistry and immunofluorescence staining

For immunohistochemistry staining of the brain sections, mice were perfused with 4% paraformaldehyde (PFA) in 10 mM phosphate-buffered saline (PBS). The brains were removed and fixed overnight in 4% PFA at 4 °C. Coronal sections (25 μm) were collected on a vibratome (Leica, Nussloch, Germany; S100, TPI) and placed in PBS with 0.1% sodium azide. Immunohistochemistry was performed on brain sections by incubating free-floating sections with primary antibodies overnight at 4 °C. The sections were further incubated with biotin-labeled secondary antibodies for 1 h at 37 °C and visualized with the DAB tetrachloride system. The images were taken using a microscope (Olympus BX60, Tokyo, Japan) at identical settings for each of the conditions. Quantitative analyses were carried out by an experimenter blind to treatment groups using an image analysis system (Image-Pro plus 6.0, ipwin32, American).

For cell line studies, SK-N-SH cells were plated on diametric glass coverslips coated with poly-D-lysine (10 μg/ml) in 24-well culture plates. After treatment with ephrinB1/Fc or Fc control, cell culture medium was carefully removed. After two rinses with PBS, the cells were fixed in a freshly prepared solution of 4% paraformaldehyde for 15 min. After two more rinses in PBS, the cells were permeabilized in 1% Triton X-100 in PBS for 15 min. Then the cells were incubated in 3% BSA in PBS for 1 h and then incubated with primary antibody at 4 °C overnight. The immunoreactivity was probed using Oregon Green 488-conjugated secondary antibodies (1:1,000; Molecular Probes) for 2 h at room temperature. The immunofluorescence images from mouse brains or cells were taken by using a confocal laser scanning microscope (FV500; Olympus, Tokyo, Japan).

### Western blotting

The cells or the hippocampal tissues were rinsed twice in ice-cold PBS (pH 7.5) and lysed with buffer containing 10 mM Tris-Cl, pH 7.6, 50 mM NaF, 1 mM Na_3_VO_4_, 1 mM edetic acid, 1 mM benzamidine, 1 mM PMSF, and a mixture of aprotinin, leupeptin, and pepstatin A (10 μg/ml each). After measurement of protein concentration in the extracts using BCA kit (Pierce, Rockford, IL), a final concentration of 10% β-mercaptoethanol and 0.05% bromophenol blue were added, and the samples were boiled for 10 min in a water bath for Western blotting^14^. The proteins in the extracts were separated by 10% SDS-PAGE and transferred to nitrocellulose membrane. The membrane was then blocked in 5% non-fat milk for 1 hr at room temperature. The membranes were incubated with primary antibody at 4 °C for overnight. Then the blots were incubated with anti-rabbit or anti-Goat IgG conjugated to IRDye™ (800CW) (1:10000) for 1 h at room temperature. Immunoreactive bands were visualized using the Odyssey Infrared Imaging System (Licor biosciences, Lincoln, NE)^28^. To eliminate the variations due to protein quantity and quality, the data were adjusted to DM1A expression (IOD of objective protein *versus* IOD of DM1A protein).

### GSK-3β activity assay

Activity of GSK-3β was measured using GSK-3β Activity Assay Kit (GENMED Scientifics INC., ARLINGTON, MA) by following the manufacturer’s manufacture’s instructions. The principle of the colorimetric method is based on the activity of GSK-3β to phosphorylate the target sequence GPHRSTPESRAAV in the presence of ATP and then through reactions that involve pyruvate kinase and lactate dehydrogenase to oxidize NADH (reduced nicotinamide adenine dinucleotide) into NAD (nicotinamide adenine dinucleotide), and the absorbance was read at 340 nm. In this assay, the GSK-3α activity was inhibited by Aloisine A (a GSK-3α inhibitor provided in the kit).

### Statistical analysis

Data were evaluated as the mean and standard deviation (mean ± SD). Statistical analysis of the quantitative multiple group comparisons was performed using the one-way analysis of variance (ANOVA) followed by Duncan’s test; whereas pairwise comparisons were performed using the t test by SPSS software (ver. 12.0; SPSS, Chicago, IL, USA). Results were considered to be statistically significant with *P* < 0.05.

## Additional Information

**How to cite this article**: Jiang, J. *et al.* Stimulation of EphB2 attenuates tau phosphorylation through PI3K/Akt-mediated inactivation of glycogen synthase kinase-3β. *Sci. Rep.* **5**, 11765; doi: 10.1038/srep11765 (2015).

## Supplementary Material

Supplementary Information

## Figures and Tables

**Table 1:** Antibodies employed in this study.

**Antibody**	**Specificity**	**Type**	**Dilution**	**Company**
EphB2		Mono-	1:1000 for WB	R&D
		Poly-	1:200 for IH & IP	Abcam
Flag		Mono-	1:1000 for WB	Millipore
Total-PI3K	Total PI3K	Poly-	1:1000 for WB	Cell Signaling
Tyr458/Tyr199- PI3K	Phospho-PI3K at Tyr458/Tyr199	Poly-	1:1000 for WB	Cell Signaling
p- PI3K-P85	Phospho-PI3K at Tyr458	Poly-	1:1000 for WB	Cell Signaling
Total-Akt	Total Akt	Poly-	1:1000 for WB	Cell Signaling
Ser473-Akt	Phospho-Akt at Ser473	Poly-	1:1000 for WB	Cell Signaling
Thr308-Akt	Phospho-Akt at Thr308	Poly-	1:1000 for WB	Abcam
Total-GSK3	Total GSK-3	Poly-	1:1000 for WB	Signalway antibody
Tyr279/Tyr216-GSK3β	Phospho- GSK3β at Tyr279/Tyr216	Mono-	1:1000 for WB	Upstate
Ser9- GSK3β	Phospho- GSK3β at Ser9	Poly-	1:1000 for WB	Cell Signaling
Tau1	Tau at Ser198/199/202	Mono-	1:1000 for WB	Chemicon
Thr205	Phospho-Tau at Thr205	Poly-	1:1000 for WB	Biosource
Thr231	Phospho-Tau at Thr231	Poly-	1:1000 for WB	Biosource
			1:500 for IF	
Ser396	Phospho-Tau at Ser396	Poly-	1:1000 for WB	Biosource
			1:500 for IF	
PY99	Phospho-Tyrosine	Mono-	1:500 for WB	Santa Cruz
DM1A	α-Tubulin	Mono-	1:1000 for WB	Sigma
Oregon green 488			1:1000 for IF	Molecular Probes
Goat anti-rabbit Ig-G (H + L)				

Mono: monoclonal, poly: polyclonal, WB: Western blotting, IF: immunofluorescence, IH: immunohistochemistry, IP: immunoprecipitation.
