# Supplementary material for: Stimulation of EphB2 attenuates tau phosphorylation through PI3K/Akt-mediated inactivation of glycogen synthase kinase-3β
Source: Sci Rep. 2015 Jun 29;5:11765. doi: 10.1038/srep11765 (PMC4484244; doi:10.1038/srep11765)
Supplement: Supplementary Information [file srep11765-s1.doc]

**Stimulation of EphB2 attenuates tau phosphorylation through PI3K/Akt-mediated** **inactivation of glycogen synthase kinase-3β**

Jun Jiang^a,c #^, Zhi-Hao Wang ^a, #^, Min Qu^a,b, *^, Di Gao^a^, Xiu-Ping Liu^a,d^, Ling-Qiang Zhu^a^, Jian-Zhi Wang^a,e,*^

^a^ Department of Pathophysiology, School of Basic Medicine and the Collaborative Innovation Center for Brain Science, Key Laboratory of Neurological Diseases of Education Ministry of China, Tongji Medical College, Huazhong University of Science and Technology, Wuhan, P.R. China

^b^ Hubei Provincial Center for Disease Control and Prevention, Wuhan, P. R. China

^c^ Department of Oncology, The Central Hospital of Wuhan, 430014, Wuhan, China.

^d^ Clinical Laboratory of Hangzhou Traditional Chinese Medical Hospital, Hangzhou, P. R. China

^e^ Co-innovation Center of Neuroregeneration, Nantong University, Nantong, JS 226001, China

*Corresponding author: Dr. Jian-Zhi Wang, email: [wangjz@mails.tjmu.edu.cn](mailto:wangjz@mails.tjmu.edu.cn), Tel.: 086-27-83692625; Fax: 0862783693883; and Dr. Min Qu, email: [minqu2977@163.com](mailto:minqu2977@163.com), Tel., 0862787652017.

# These authors contributed equally


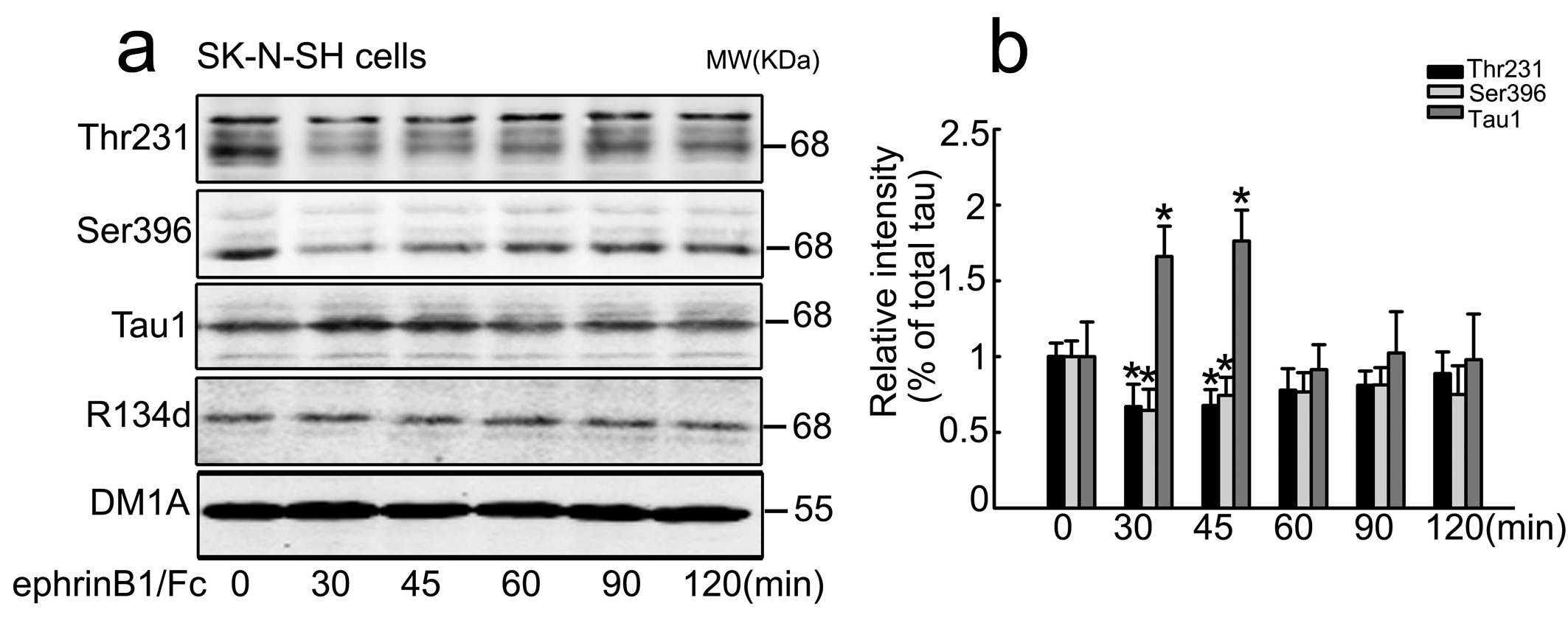


**Supplementary Fig 1 | Stimulation of EphB2 by ephrinB1/Fc induces a transient dephosphorylation of tau.** The SK-N-SH cells were treated with ephrinB1/Fc, a chimeric stimulator of EphB2, for different times as indicated and then tau phosphorylation was measured by Western blotting (a) and quantitative analysis (b) using a panel of phosphorylation-dependent antibodies [Note that tau-1 reacts with the unphosphorylated tau at Ser198/199/202, and an increased immunoreaction to tau-1 suggests an increased tau dephosphorylation]. Level of tau phosphorylation was normalized against total tau probed by R134d, and the total tau level was normalized against tubulin probed by DM1A. *P<0.05 versus 0 min.

 
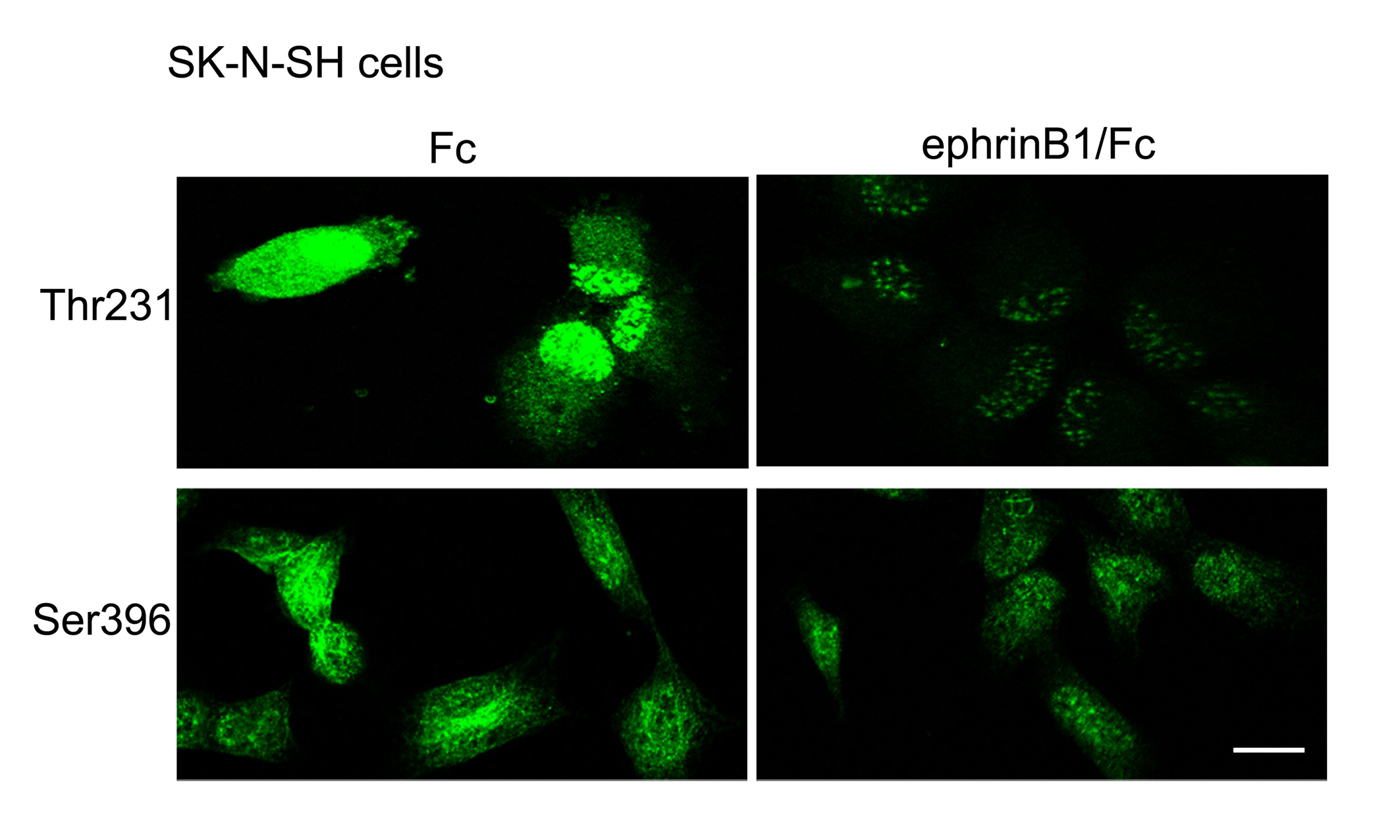


**Supplementary Fig 2 | Stimulation of EphB2 by ephrinB1/Fc for 30 min induces dephosphorylation of tau at Thr231 and Ser396 in SK-N-SH measured by immunofluorescence staining.** Scale bar =5 μm.

**
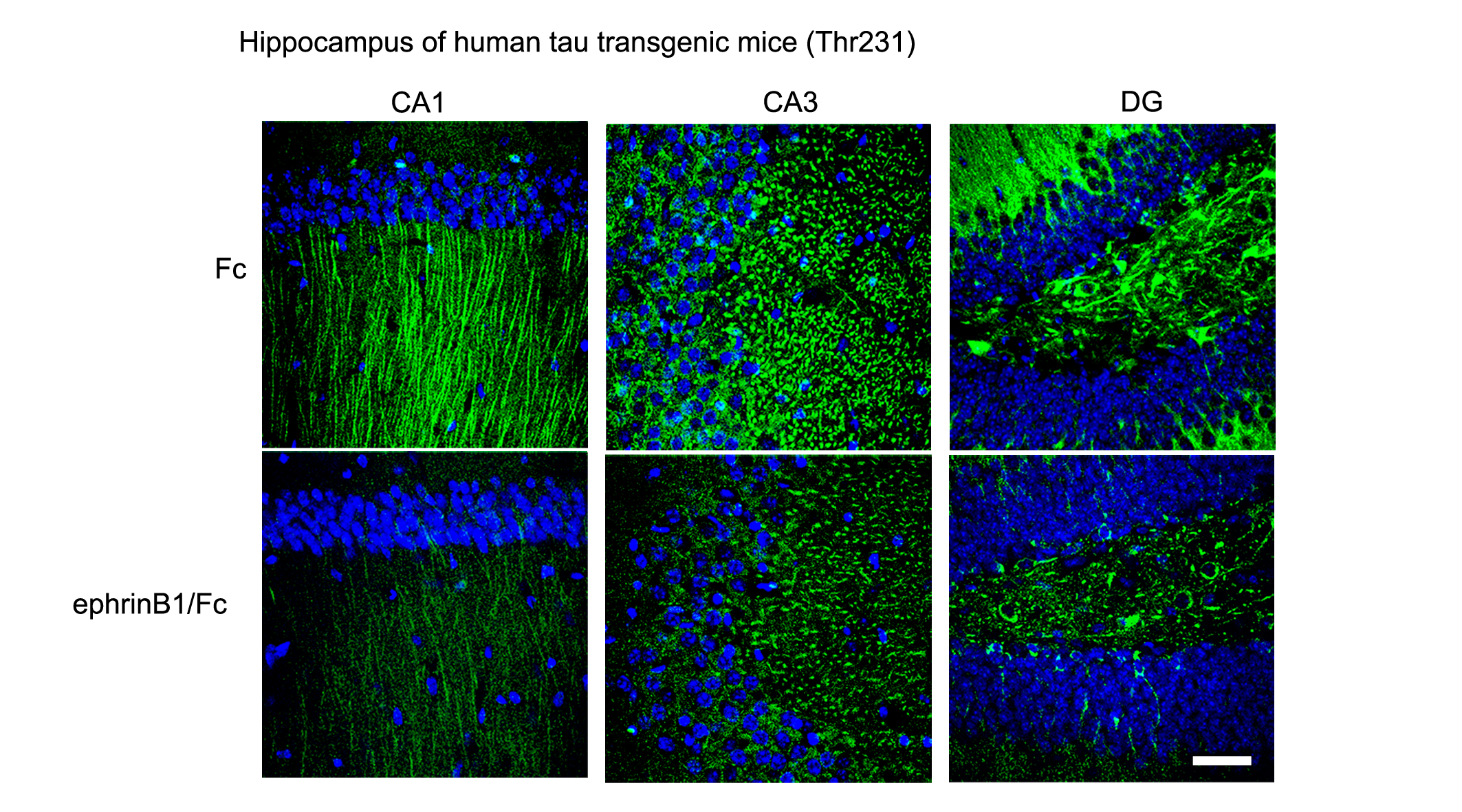
** **Supplementary Fig 3 | Stimulation of EphB2 induces tau dephosphorylation in hippocampus of human tau transgenic mice.** The ephrinB1/Fc was infused into hippocampal CA3 region of the human tau transgenic mice (10m old) for 45 min, and then tau phosphorylation in hippocampus was measured by immunofluorescence staining. Scale bar = 20 μm.
